# Molecular Dynamics Simulation of Silane Inserted CSH Nanostructure

**DOI:** 10.3390/ma17010149

**Published:** 2023-12-27

**Authors:** Fei Yang, Yangyang Cui, Anming She, Ran Hai, Zheyu Zhu

**Affiliations:** 1 School of Architectural and Civil Engineering, Zhongyuan University of Technology, Zhengzhou 450007, China; 2021109332@zut.edu.cn (Y.C.); lilyhai_2001@163.com (R.H.); 2 Key Laboratory of Advanced Civil Engineering Materials of Ministry of Education, School of Materials Science and Engineering, Tongji University, Shanghai 201804, China; sheanming@tongji.edu.cn; 3 School of Materials Science and Engineering, Yancheng Institute of Technology, Yancheng 224051, China; 17712937215@163.com

**Keywords:** molecular dynamics simulation, calcium silicate hydrate, tensile property, mean azimuth shift

## Abstract

Herein, the toughening mechanism and effects of 3-(aminopropyl)triethoxysilane (3-APTES) intercalation in calcium–silicate–hydrate (CSH) structures were investigated through molecular dynamics simulations. CSH established a model using 11 Å-tobermorite to simulate the tensile properties, toughness, adsorption energy, average orientation displacement and radial distribution function of 3-APTES intercalation at different Ca/Si ratios under conditions of a CVFF force field, an NVT system, and 298 K temperature. Simulation results demonstrate that 3-APTES alters the fracture process of CSH and effectively enhances its tensile properties and toughness. The presence of 3-APTES molecules increases the energy required to destroy CSH, thereby increasing the adsorption energy of CSH crystals. Furthermore, 3-APTES molecules effectively increase the atom density within the CSH structure. As the Ca/Si ratio increases, Ca–O bond formation is enhanced, with noticeable aggregation occurring because of modification by 3-APTES within the CSH structure. This study found that 3-APTES organic compounds can effectively improve the tensile, toughness, adsorption and other properties of the CSH structure, and further improve the microstructure of CSH.

## 1. Introduction

The hydration reaction of Portland cement upon contact with water leads to various products such as calcium–silicate–hydrate (CSH), calcium hydroxide, and ettringite. CSH constitutes approximately 60% of the total mass of cement slurry and is crucial for determining the mechanical properties, such as strength and durability, of cement-based materials. CSH gels possess intricate multiscale porous structures comprising nanopores and larger pores. The transport of Ca ions and water molecules within CSH nanopores notably characterizes the strength, shrinkage, creep, and physicochemical reaction properties of materials [1]. Under the influence of applied loads and environmental factors, CSH continuously changes physically and chemically, thereby inducing molecular structure defects within CSH that weaken bonding forces in cement-based materials, ultimately resulting in structural damage. The CSH structure model resembles tobermorite or jennite structures comprising a Ca–O skeleton with Si chains on both sides. Studying tobermorite or jennite structures can facilitate the CSH structure model optimization [2,3].

Recently, polymer-modified CSH in concrete and mortar has been widely used to enhance the structural properties of cement-based materials. Organic modification of CSH involves a hybrid CSH synthesis by precipitating a combination of Ca salt and organic matter in an aqueous solution. This process helps preserve the inorganic Ca–Si layered structure while incorporating interlayer organic groups [4]. The organic CSH intercalation method involves embedding polymers between calcium oxide and silica tetrahedral layers in CSH. Matsuyama [5] was the first to combine negative and cationic polymers with CSH and observed that intercalating macromolecules between inorganic layers could increase interlayer spacing. Embedding polymers such as polyvinyl alcohol, polyethylene glycol, and polyaniline into the CSH interlayer can improve its toughness, creep resistance, corrosion resistance, and volume stability [6]. Jennings [7] proposed a nanoscale gel model that suggests that the smallest structural unit in the CSH gel is formed by stacking 2.2 nm particles, resulting in a CSH size of 5.6 nm. Therefore, studying the microstructure of polymer intercalation in CSH is considerably important.

Silanes are well-known for their ability to effectively connect inorganic and organic substrates [8,9,10,11]. Silane molecules possess “Si–O–R” bonds, with the Si–O bond playing a crucial bridging role in connecting with inorganic materials within the CSH matrix [12]. Experimentally, 3-(aminopropyl)triethoxysilane (3-APTES) can be intercalated into CSH layers, resulting in the formation of unique structures where 3-APTES is intercalated within CSH. Theoretically, this intercalation mechanism can enhance the molecular-scale toughness of CSH. However, to the best of our knowledge, research on the investigation of the structural properties of CSH and the toughening mechanism of 3-APTES intercalation is inadequate [13,14,15]. Anelia et al. [16] studied the effects of graphene on the growth of AIN and found that graphene promoted the decomposition of (CH3) 3AL and formed AL adsorbed atoms. At the same time, the high deposition temperature also ensured the sufficient diffusion of AL adsorbed atoms, thereby improving the quality of the material; GDS et al. [17] simulated the collision process between TMIn and hydrogen atoms, and studied the transfer of protons between the intramolecular and surface protons. They also investigated how TMIn directly reacts with graphene to achieve TMIn conversion, ultimately obtaining the transfer of IN monomers or INH and CH3IN molecules on graphene.

Herein, we investigated the toughening mechanism and effect of 3-APTES intercalation in CSH structures through molecular dynamics simulations. We calculated the stress–strain curve and specific volume strain energy of CSH. The adsorption energy between water molecules and silicate tetrahedra before and after 3-APTES intercalation was examined. In addition, changes in Ca–O bonds and pore diffusion states were observed during the tensile process before and after the 3-APTES intercalation. This study provides theoretical references for synthesizing novel high-toughness and high-strength silicate cement.

## 2. Simulation Method

### 2.1. Models

**Figure 1:**
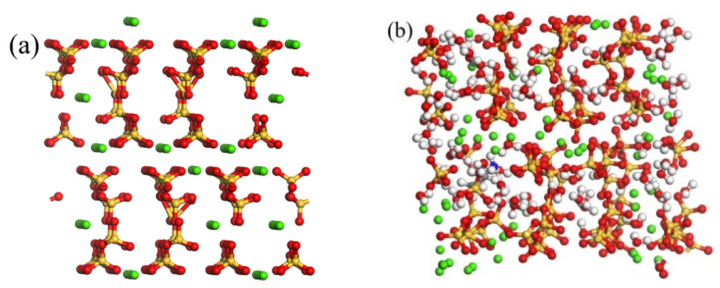
The CSH crystal models: (**a**) expanded structure; (**b**) filled with water molecules.

Based on the 11 Å-tobermorite as the reference structure, we constructed the crystal structure of the CSH gel [18,19] (see Figure 1).

In Figure 1, green represents Ca, red represents O, and yellow represents Si. The ratio between calcium and silicon atoms in the CSH structure is called the calcium–silicon ratio, which is Ca/Si. By removing silicate tetrahedra and calcium ions, the Ca/Si ratio was adjusted to 0.83 and 1.7, satisfying the Q^n^ distribution observed in solid-state nuclear magnetic resonance (NMR) studies and consistent with most experimental investigations on CSH. When Ca/Si is 0.83, the contents of Q^0^, Q^1^, Q^2^, and Q^3^ are 4.1%, 10.9%, 77.4%, and 7.7%, respectively; and when Ca/Si is 1.7, the contents of Q^1^, Q^2^, and Q^3^ are 16.9%, 70.1%, and 13%, respectively. The structures were then expanded and water molecules were added using the grand canonical Monte Carlo (GCMC) method at 298 K and 1 g/cm^3^ until the CSH crystal achieved 100% water saturation [20,21,22]. Finally, 3-APTES was inserted into the CSH crystal structures with different Ca/Si ratios. The pure CSH model and the 3-APTES modified CSH model were subjected to energy optimization using the CVFF force field and then molecular dynamics simulations were performed under the NVT ensemble [23].

### 2.2. Force Field

The CVFF force field is commonly used for structure, energy, and dynamics simulations of polymers such as proteins, peptides, and other organic systems, and has been proven to be highly suitable for simulating organic crystals like amides and carboxylic acids [24]. The CVFF force field also describes the interactions between crystals and parameters such as bond lengths, bond angles, and torsional angles. Therefore, in this study, the CVFF force field was employed to handle the organic polymer atoms and facilitate the molecular dynamics simulations. The simulations were conducted using LAMMPS software (https://www.lammps.org/, accessed on 4 December 2023), with the CVFF force field applied to each model system for energy minimization and subsequently for molecular dynamics until equilibrium was achieved. The simulations were performed in the NVT ensemble at a temperature of 298 K [25,26,27,28]. To ensure sufficient interaction between the polymer and the CSH, an equilibration time of 4 ns was chosen, during which the trajectories of all atoms were collected for further structural and dynamic analysis.

When conducting uniaxial tensile tests on the CSH models, the polymer–CSH crystal interaction was examined under different calcium-to-silicon ratios. The NPT ensemble method was utilized to release any excess stress, ensuring that the model was not subjected to external pressure in the x-, y-, and z-axis directions. The 3-APTES modified CSH model was subjected to a tensile rate of 0.08 ps-1 in the x-, y-, and z-axis directions. When the model was stretched along a specific axis, the forces acting in the other two orthogonal directions were set to zero, indicating that the applied force was the principal stress in the stretching direction.

During toughness tests on the CSH models, the material’s toughness variations were primarily characterized by the stress and strain of the material. The strain energy per unit volume was utilized to evaluate the toughness changes in the 3-APTES modified CSH models. The strain energy per unit volume represents the potential energy of stress and strain within the material [29,30,31,32]. The formula for calculating the strain energy per unit volume is as follows:

$$\mathrm{En}=\int_{\varepsilon 0}^{\varepsilon ƒ}\sigma (\varepsilon )d\varepsilon$$

where En represents the strain energy per unit volume, σ is stress, and ε is strain.

## 3. Molecular Dynamics Simulation Assessment

### 3.1. Tensile Mechanical Properties

**Figure 2:**
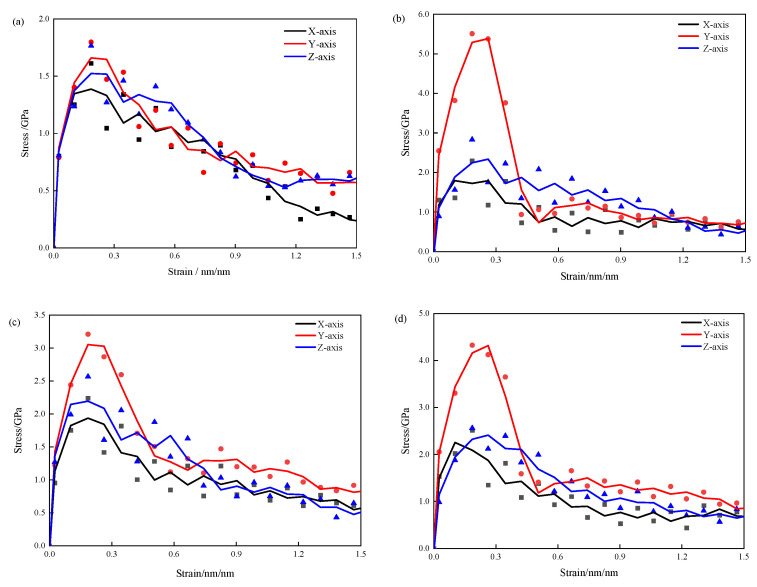
Tensile stress–strain curves of CSH: (**a**) pure CSH with Ca/Si ratio of 0.83; (**b**) pure CSH with Ca/Si ratio of 1.70; (**c**) modified CSH with Ca/Si ratio of 0.83; (**d**) modified CSH with Ca/Si ratio of 1.70.

Figure 2 illustrates the stress–strain curves of pure CSH and 3-APTES modified CSH under conditions of a Ca/Si ratio of 0.83 and a Ca/Si ratio of 1.7. In Figure 2, it is evident that the 3-APTES modified CSH exhibits better plasticity and strain variables compared to the pure CSH model when the tensile direction is along the y-axis.

This indicates that the y-axis tensile behavior is parallel to the direction of CSH single chains, where multiple CSH single chains are formed after the expansion of the CSH unit cell. As a result, the elastic and yield stages of the y-axis tensile behavior show significant improvements compared to the x-axis and z-axis.

As seen in Figure 2a,b, under the condition of a Ca/Si ratio of 0.83, the elastic stages of the 3-APTES modified CSH model are consistently superior to the pure CSH model in all three directions. The maximum tensile stresses during the elastic stage of the organically modified CSH are significantly increased, with the maximum stress along the y-axis being approximately 5.0–5.5 GPa. This indicates that 3-APTES organic modification can effectively enhance the yield strength and improve the ductility of the CSH material. 

Under the condition of a Ca/Si ratio of 1.7, the elastic and yield stages of the 3-APTES modified CSH, when stretched along the y-axis, are significantly higher than the other two axes. The changes in tensile performance between the 3-APTES modified CSH and pure CSH along the x-axis and z-axis are less pronounced. This indicates that regardless of the Ca/Si ratio, organic modification of 3-APTES can improve the tensile properties of CSH. This is mainly because after 3-APTES organic modification of CSH, the oxygen and hydrogen atoms in the original silicate chain break and combine with the broken hydrogen atoms in the 3-APTES organic compound to form new molecules, which undergo linking reactions and improve the material’s ductility.

In conclusion, the tensile performance tests of CSH were conducted along the x-, y-, and z-axes. As seen in Figure 2, with an increasing Ca/Si ratio, the maximum tensile stress of the pure CSH model increases, while the maximum tensile stress of the high-Ca/Si ratio 3-APTES modified CSH model decreases compared to the low-Ca/Si ratio one. However, the tensile performance of the 3-APTES modified model is superior to the pure CSH model in all cases. This primarily indicates that the increase in the Ca/Si ratio reduces the corresponding water molecules, which contribute to the bonding performance. As a result, the maximum tensile stress shows a decreasing trend. The simulation results are consistent with relevant experimental findings.

### 3.2. Toughness Performance

**Figure 3:**
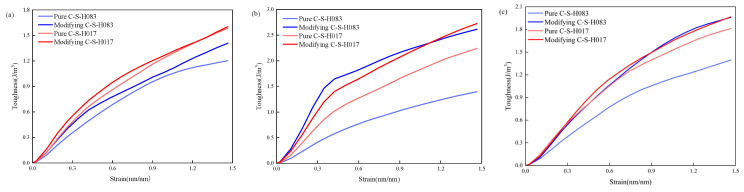
Volume energy of CSH modified by pure CSH and 3-APTES with Ca/Si ratios of 0.83 and 1.7: (**a**) the x-axis; (**b**) y-axis; (**c**) z-axis.

Figure 3 shows the specific volumetric strain energy (En) of pure CSH with Ca/Si ratios of 0.83 and 1.7, as well as 3-APTES modified CSH.

**Figure 4:**
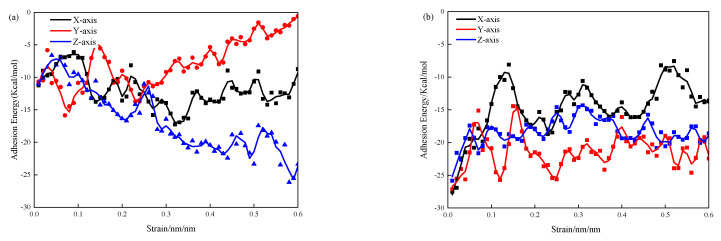
Adsorption properties of 3-APTES modified CSH model: (**a**) Ca/Si ratio of 0.83; (**b**) Ca/Si ratio of 1.7.

In Figure 4, it can be observed that the En values of the 3-APTES modified CSH models with Ca/Si ratios of 0.83 and 1.7 are higher than those of the pure CSH models.

Specifically, for the Ca/Si ratio of 0.83, this indicates that the 3-APTES modified CSH model exhibits improved toughness. For the Ca/Si ratio of 0.83, the specific volumetric strain energies (En) of the pure CSH model are 1.20 J/m^3^, 1.40 J/m^3^, and 1.39 J/m^3^ along the x-, y-, and z-axes, respectively. On the other hand, the 3-APTES modified CSH model exhibits En values of 1.41 J/m^3^, 2.61 J/m^3^, and 1.96 J/m3 along the x-, y-, and z-axes, respectively. For the Ca/Si ratio of 1.7, the pure CSH model has En values of 1.58 J/m^3^, 2.24 J/m^3^, and 1.81 J/m^3^ along the x-, y-, and z-axes, respectively. Meanwhile, the 3-APTES modified CSH model has En values of 1.60 J/m3, 2.73 J/m^3^, and 1.97 J/m^3^ along the x-, y-, and z-axes, respectively. Along the x-axis, the strain energy (En) values of the 3-APTES modified CSH with Ca/Si ratios of 0.83 and 1.7 increased by 17.5% and 1.3%, respectively. Along the y-axis, the 3-APTES modified CSH with Ca/Si ratios of 0.83 and 1.7 exhibited En values that increased by 86.4% and 21.9%, respectively. Similarly, along the z-axis, the 3-APTES modified CSH with Ca/Si ratios of 0.83 and 1.7 showed En values that increased by 41.0% and 8.8%, respectively. Furthermore, for both Ca/Si ratios of 0.83 and 1.7, the pure CSH models and the 3-APTES modified CSH models exhibited overlapping elastic deformation stages, indicating similar toughness during this stage. However, in the plastic deformation stage, the 3-APTES modified CSH models displayed better toughness compared to the pure CSH models.

As seen in Figure 3, the strain energy (En) values of both the pure CSH and 3-APTES modified CSH models with a Ca/Si ratio of 1.7 are higher than those of the pure CSH and 3-APTES modified CSH models with a Ca/Si ratio of 0.83. This indicates that as the Ca/Si ratio increases, the average silicate chain length decreases while the interlayer water molecules increase. Subsequently, the insertion of the 3-APTES organic compound enhances the En values of the CSH model, thereby improving the toughness and deformability of the CSH model. Based on this, under the same Ca/Si ratio, the tensile properties and toughness of the 3-APTES modified CSH structure are significantly improved compared to the pure CSH structure. Additionally, by increasing the Ca/Si ratio of the CSH model, not only the tensile properties of the pure CSH structure are enhanced, but the toughness of the material is also effectively improved. However, the tensile properties of the 3-APTES modified CSH structure decrease with increasing Ca/Si ratio, while the toughness performance increases with the increasing Ca/Si ratio.

### 3.3. Adsorption Energy

Figure 4 illustrates the adsorption performance between water molecules and the silicon chain in the 3-APTES modified CSH model under different calcium–silicon ratios. As seen in Figure 4a, the tensile adsorption performance along the y-axis increases with the increasing strain, while the adsorption performance along the x-axis and z-axis decreases relatively with the increasing strain. At a strain of 0.6 nm/nm, the adsorption energy along the y-axis reaches as high as −0.56 kcal/mol. This indicates that when stretched along the distribution direction of the silicon chain, there is stronger bonding between the silicon chains and a lower amount of water molecule distribution. As the strain increases, the adsorption performance enhances. When stretched perpendicular to the silicon chain direction, there are more water molecules distributed, leading to a continuous decrease in the adsorption capacity, which suggests that the stretching direction has a significant impact on the adsorption energy between the interface and water molecules. As seen in Figure 4b, when conducting stretching tests in different directions in the CSH simulation, the adsorption capacity between the interface and water molecules strengthens with the increasing strain. With the increase of the calcium–silicon ratio, the 3-APTES organic compound exhibits higher deformation ability, and water molecules are distributed relatively uniformly in all directions, thereby enhancing the adsorption capacity between the crystal interface and water molecules.

As the calcium–silicon ratio increases in Figure 4, the adsorption capacity decreases continuously with the increasing strain. The increase in the calcium–silicon ratio leads to an increase in the number of interlayer water molecules, which reduces the overall adsorption energy of the CSH crystal. During the elastic deformation of the CSH, a small number of pores may form in the intermediate layer and the interior of the CSH crystal. The deformation of the 3-APTES molecules helps counteract the energy generated during the stretching process. Therefore, the 3-APTES molecules enhance the energy required for CSH fracture, thereby improving the adsorption capacity of the CSH crystal.

### 3.4. Mean Square Displacement

**Figure 5:**
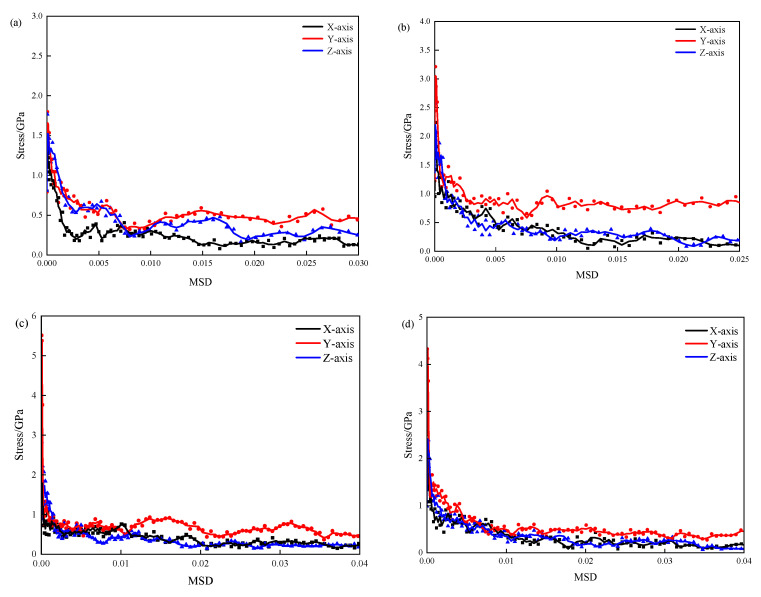
Mean square displacement curves: (**a**) pure CSH with Ca/Si ratio of 0.83; (**b**) pure CSH with Ca/Si ratio of 1.7; (**c**) modified CSH with Ca/Si ratio of 0.83; (**d**) modified CSH with Ca/Si ratio of 1.7.

Figure 5 shows the stress versus total mean square displacement (MSD) curves for the pure CSH and 3-APTES modified models at calcium–silicon ratios of 0.83 and 1.7.

**Figure 6:**
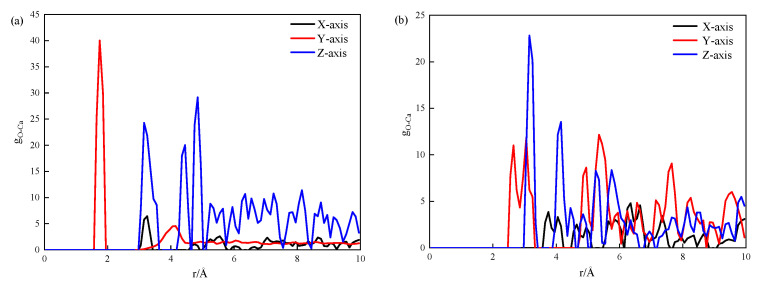
RDF of Ca-O bonds: (**a**) Ca/Si ratio of 0.83; (**b**) Ca/Si ratio of 1.7.

The MSD represents the trend of water molecules within the crystal structure as the stress changes. From Figure 6, it can be observed that as the MSD increases, the stress exhibits a sudden drop during the elastic stage, followed by a slow decreasing trend.

Specifically, we found that MSD (y-axis) > MSD (z-axis) > MSD (x-axis). During the elastic stage, ionic bonds such as Si-O bonds and H-O bonds do not rupture. As the stretching time continues, the tensile stress increases, leading to the formation of cracks between the ionic bonds. At the yield point, there is a sudden change in MSD due to the appearance of cracks, resulting in a drastic drop in stress. With the progression of the tensile test, the MSD continues to increase. The required tensile stress for causing mean square displacement along the vertical direction is smaller than that in the direction parallel to the silicon chains, which explains the higher stress observed on the y-axis when MSD is the same.

As seen in Figure 5a–d, after the modification of CSH with 3-APTES, the stress required for the 3-APTES modified CSH model is higher than that for the pure CSH model at the same MSD value. Conversely, when the stress values are the same, the MSD value decreases for the 3-APTES modified CSH model. In the pure CSH model during stretching, as time increases, internal atoms such as Ca and Si continuously diffuse, resulting in an increasing MSD value. However, after organic modification with 3-APTES, the 3-APTES silane, which has double bonds, enhances the bonding performance between CSH, and increases the density between atoms. This leads to a relatively smaller MSD value for the 3-APTES modified CSH model compared to the pure CSH model under the same stress conditions.

### 3.5. Radial Distribution Function

The radial distribution function (RDF) is a function that describes the density variation of particles within a certain distance r from a target atom. It can reflect the probability and distribution pattern of the occurrence of a certain particle within a specified distance in a structural model. RDF is a common analytical method used in molecular dynamics simulations.

Figure 6 displays the RDF of Ca-O in the 3-APTES organic matter and CSH models at different calcium–silicon ratios.

In Figure 6a, it can be observed that when the modified CSH is stretched along the x-, y-, and z-axes, the maximum peak positions of Ca-O are within the ranges of 3.3–3.5 Å, 1.8–2 Å, and 4.3–4.5 Å, respectively. This indicates that when the modified CSH is stretched along the x-, y-, and z-axes, the number of Ca-O chains at 2 Å, 3.5 Å, and 4.5 Å increases, leading to a significant increase in density. In Figure 6b, it can be seen that when the modified CSH is stretched along the x-, y-, and z-axes, the maximum peak positions of Ca-O are within the ranges of 6.2–6.4 Å, 5.2–5.4 Å, and 3.2–3.4 Å, respectively. This suggests that when the modified CSH is stretched along the x-, y-, and z-axes, the most significant density enhancement occurs at 6.4 Å, 5.4 Å, and 3.4 Å, respectively. With an increase in the Ca/Si ratio, the RDF values continuously decrease, and the r values between atoms increase. This indicates that as the Ca/Si ratio increases, the number of Ca-O bonds increases. There is bonding between the calcium and oxygen atoms, leading to a noticeable aggregation of Ca-O after the 3-APTES modification.

### 3.6. Tensile Dynamic Analysis

**Figure 7:**
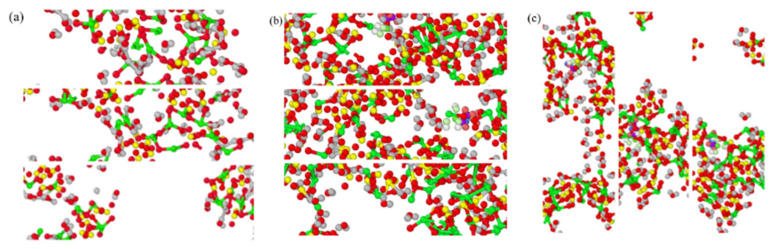

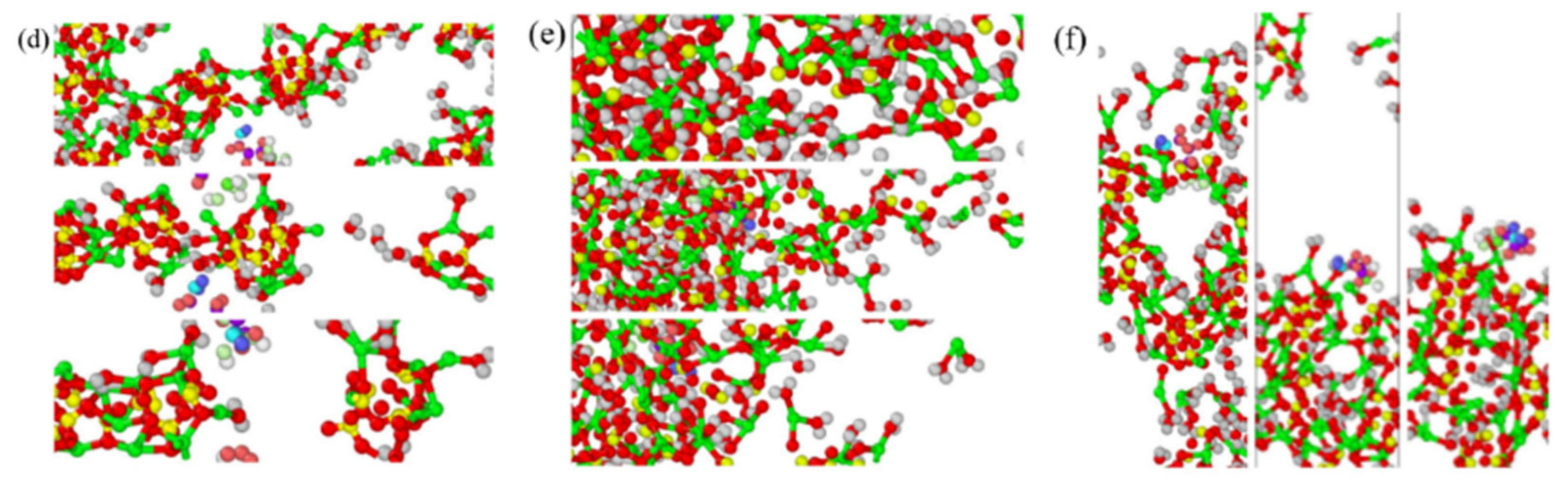
3-APTES modified CSH tensile dynamic network: (**a**) Ca/Si ratio of 0.83 along the x-axis; (**b**) Ca/Si ratio of 0.83 along the y-axis; (**c**) Ca/Si ratio of 0.83 along the z-axis; (**d**) Ca/Si ratio of 1.7 along the x-axis; (**e**) Ca/Si ratio of 1.7 along the y-axis; (**f**) Ca/Si ratio of 1.7 along the z-axis.

Figure 7 illustrates the dynamic network diagrams of 3-APTES modified CSH crystals under various calcium–silicon ratios during stretching along different directions.

The figure displays the elastic stage, yield stage, and fracture stage of the 3-APTES modified CSH crystal during the stretching process [33,34]. Cracking patterns are observed in the elastic stage during stretching along all directions. As the stretching experiment progresses, the cracks gradually widen, leading to the yield stage until the structure reaches its maximum yield stress. As seen in Figure 7b, during the elastic stage of 3-APTES structure stretching along the y-axis, a significant aggregation of silicate tetrahedra and water molecules is observed. This is primarily attributed to the presence of hydrogen bonding in the 3-APTES organic compound, which effectively adsorbs O from silicate tetrahedra and water molecules, thereby enhancing the adsorption capacity within the CSH crystal structure [35,36,37]. In Figure 7c,f, it can be observed that cracks have already appeared in the elastic stage of the 3-APTES modified CSH crystal, and there is no significant difference between the yield stage and the fracture stage. This is mainly due to the water molecules filling the space between the upper and lower layers of silicate chains, providing the corresponding internal force during vertical stretching. The intercalation of the 3-APTES organic material is parallel to the direction of the silicate chain, resulting in a lower vertical limit tensile stress it can withstand. As a result, large cracks appear in the elastic stage, exhibiting similar phenomena in the yield stage and the fracture stage.

By comparing Figure 7a and Figure 7d, Figure 7b and Figure 7e, and Figure 7c and Figure 7f, it is evident that all stages of stretching for the 3-APTES modified CSH crystal with a calcium–silicon ratio of 1.7 outperform those with a ratio of 0.83. This is primarily because as the calcium–silicon ratio increases, there are less water molecules filling the space, allowing for the formation of more bonding groups such as Ca-O, Si-O, and H-O within the internal structure. This enhances the cohesion of the structure and strengthens its resistance to stress from various directions, thus improving the mechanical performance of the CSH structure.

## 4. Conclusions

The structure of CSH intercalated by 3-APTES was studied using molecular dynamics simulations, and the differences in the interaction mechanism between 3-APTES modified and pure CSH structures with different Ca/Si ratios were elucidated. The overall conclusions of this study are as follows:

1. The 3-APTES modified CSH structure exhibits better tensile properties than the pure CSH structure. Compared with the pure CSH structure, the maximum tensile stress of the 3-APTES modified CSH structure along the x-, y-, and z-axes increases by 45.4%, 78.6%, and 38.9%, respectively, at a 0.83 Ca/Si ratio. Moreover, at a 1.7 Ca/Si ratio, the maximum tensile stress along the x-, y-, and z-axes increases by 13.5%, 63.3%, and 16.6%, respectively.
2. The toughness of the 3-APTES modified CSH structure is superior to that of the pure CSH structure. At a 0.83 Ca/Si ratio, the En values of the 3-APTES modified CSH structure along the x-, y-, and z-axes increase by 17.5%, 86.4%, and 41.0%, respectively, compared to the pure CSH structure. Moreover, at a 1.7 Ca/Si ratio, the En values along the x-, y-, and z-axes increase by 1.3%, 21.9%, and 8.8%, respectively.
3. The incorporation of 3-APTES increases the adsorption energy of water molecules on the CSH structure model and silica tetrahedral skeleton. At a 0.83 Ca/Si ratio, the adsorption energy of the 3-APTES modified CSH structure along the x-, y-, and z-axes increases by 23.5%, 94.9%, and −97.0%, respectively, compared to the pure CSH structure. Moreover, at a 1.7 Ca/Si ratio, the adsorption energy along the x-, y-, and z-axes increases by 19.2%, 24.5%, and 25.4%, respectively. 3-APTES molecules can effectively increase the atom density within the CSH structure model. As the Ca/Si ratio increases, the Ca–O bond formation is enhanced, with noticeable aggregation occurring because of the 3-APTES-based CSH structural modification.

## Data Availability

Data are contained within the article.

## References

[1] Bonaccorsi E., Merlino S., Taylor H.F.W. (2003). The crystal structure of jennite, Ca_9_Si_6_O_18_(OH)_6_ 8H_2_O. Cem. Concr. Res..

[2] Akono A.T. (2020). Nanostructure and Fracture Behavior of Carbon Nanofiber-Reinforced Cement Using Nanoscale Depth-Sensing Methods. Materials.

[3] Zehtab B., Tarighat A. (2016). Diffusion study for chloride ions and water molecules in C-S-H gel in nano-scale using molecular dynamics: Case study of tobermorite. Adv. Concr. Constr..

[4] Plank J., Schönlein M., Kanchanason V. (2018). Study on the early crystallization of calcium silicate hydrate (C-S-H) in the presence of polycarboxylate superplasticizers. J. Organomet. Chem..

[5] Matsuyama H., Young F.J. (1999). Synthesis of calcium silicate hydrate/polymer complexes: Part I. Anionic and nonionic polymers. J. Mater. Res..

[6] Bahraq A.A., Al-Osta M.A., Obot I.B., Al-Amoudi O.S., Saleh T.A., Maslehuddin M. (2022). Improving the adhesion properties of cement/epoxy interface using graphene-based nanomaterials: Insights from molecular dynamics simulation. Cem. Concr. Comp..

[7] Jennings H.M. (2007). Refinements to colloid model of C-S-H in cement: CM-II. Cem. Concr. Res..

[8] Kamali M., Ghahremaninezhad A. (2018). Effect of Biomolecules on the Nanostructure and Nanomechanical Property of Calcium-Silicate-Hydrate. Sci. Rep..

[9] Liang G.W., Luo L., Wu Y. (2022). Reusing waste red brick powder as partial mineral precursor in eco-friendly binders: Reaction kinetics, microstructure and life-cycle assessment. Resour. Conserv. Recycl..

[10] Dal Sasso G., Dalconi M.C., Ferrari G., Pedersen J.S., Tamburini S., Bertolotti F., Guagliardi A., Bruno M., Valentini L., Artioli G. (2022). An Atomistic Model Describing the Structure and Morphology of Cu-Doped C-S-H Hardening Accelerator Nanoparticles. Nanomaterials.

[11] Murray J.S., Subramani J.V., Selvam P.R., Hall K.D. (2010). Molecular Dynamics to Understand the Mechanical Behavior of Cement Paste. Transp. Res. Rec..

[12] Bahraq A.A., Obot I., Al-Osta M.A., Al-Amoudi O.S.B., Maslehuddin M. (2022). Molecular-level investigation on the effect of surface moisture on the bonding behavior of cement-epoxy interface. J. Build. Eng..

[13] Sekkal W., Zaoui A. (2017). Enhancing the interfacial bond strength of cement nanocomposite with carbonate nanostructure. Compos. Part B—Eng..

[14] Cuesta A., Santacruz I., Angeles G., Dapiaggi M., Zea-Garcia J.D., Aranda M.A. (2021). Local structure and Ca/Si ratio in C-S-H gels from hydration of blends of tricalcium silicate and silica fume. Cem. Concr. Res..

[15] Hajilar S., Shafei B. (2015). Nano-scale investigation of elastic properties of hydrated cement paste constituents using molecular dynamics simulations. Comp. Mater. Sci..

[16] Kakanakova-Georgieva A., Ivanov I.G., Suwannaharn N., Hsu C.W., Cora I., Pécz B., Giannazzo F., Sangiovanni D.G., Gueorguiev G.K. (2021). MOCVD of AlN on epitaxial graphene at extreme temperatures. CrystEngComm.

[17] Sangiovanni D.G., Ricardo F., Kostov G.A., Kakanakova-Georgieva A. (2022). Discovering atomistic pathways for supply of metal atoms from methyl-based precursors to graphene surface. Phys. Chem. Chem. Phys..

[18] Skinner L.B., Chae S.R., Benmore C.J., Wenk H.R., Monteiro P.J.M. (2010). Nanostructure of calcium silicate hydrates in cements. Phys. Rev. Lett..

[19] Sabet A.B., Hashemi S.A.H., Farokhzad R., Delnavaz A. (2023). Synergic effect of defects on carbon nanoparticles under interaction with calcium silicate hydrate composites. Appl. Surf. Sci..

[20] Moradi M., Rezaei M. (2022). Construction of highly anti-corrosion and super-hydrophobic polypropylene/graphene oxide nanocomposite coatings on carbon steel: Experimental, electrochemical and molecular dynamics studies. Constr. Build. Mater..

[21] Mazaheripour H., Faria R., Azenha M., Ye G. (2021). Modelling Macroscopic Shrinkage of Hardened Cement Paste Considering C-S-H Densification. Adv. Cem. Res..

[22] Duque-Redondo E., Masoero E., Manzano H. (2022). Nanoscale shear cohesion between cement hydrates: The role of water diffusivity under structural and electrostatic confinement. Cem. Concr. Res..

[23] Bahraq A.A., Al-Osta M.A., Al-Amoudi O.S., Obot I.B., Adesina A.Y., Maslehuddin M. (2022). A nanoscale adhesion mechanism of cement-epoxy interface under varying moisture conditions: A molecular dynamics study. Surf. Interfaces.

[24] Geng G., Myers R.J., Li J., Maboudian R., Carraro C., Shapiro D.A., Monteiro P.J. (2017). Aluminum-induced dreierketten chain cross-links increase the mechanical properties of nanocrystalline calcium aluminosilicate hydrate. Sci. Rep..

[25] Vardhan K., Goyal S., Siddique R., Singh M. (2015). Mechanical properties and microstructural analysis of cement mortar incorporating marble powder as partial replacement of cement. Constr. Build. Mater..

[26] Liang G.W., Wu Y., She A.M. (2023). New insights into the early-age reaction kinetics of metakaolin geopolymer by 1H low-field NMR and isothermal calorimetry. Cem. Concr. Comp..

[27] Eftekhari M., Mohammadi S. (2016). Molecular dynamics simulation of the nonlinear behavior of the CNT-reinforced calcium silicate hydrate (C–S–H) composite. Compos. Part A Appl. Sci. Manuf..

[28] Khoshnazar R., Beaudoin J.J., Raki L., Alizadeh R. (2014). Volume Stability of C-S-H/Polyaniline Nanocomposites in Aqueous Salt Solutions. ACI Mater. J..

[29] Fu J., Bernard F., Kamali-Bernard S. (2018). Assessment of the elastic properties of amorphous calcium silicates hydrates (I) and (II) structures by molecular dynamics simulation. Mol. Simulat..

[30] Liang G.W., Liu T.J., Li H.R. (2022). Shrinkage mitigation, strength enhancement and microstructure improvement of alkali-activated slag/fly ash binders by ultrafine waste concrete powder. Compos. Part B—Eng..

[31] Abdolhosseini Qomi M.J., Ulm F.J., Pellenq R.J. (2012). Evidence on the Dual Nature of Aluminum in the Calcium-Silicate-Hydrates Based on Atomistic Simulations. J. Am. Ceram. Soc..

[32] Mohamed A.K., Parker S.C., Bowen P., Galmarini S. (2018). An atomistic building block description of C-S-H—Towards a realistic C-S-H model. Cem. Concr. Res..

[33] Pellenq R.J.-M., Kushima A., Shahsavari R., Van Vliet K.J., Buehler M.J., Yip S., Ulm F.-J. (2009). A realistic molecular model of cement hydrates. Proc. Natl. Acad. Sci. USA.

[34] Ali M.S., Rub M.A., Khan F., Al-Lohedan H.A. (2013). Thermodynamic, interfacial and hydrodynamic aspects of interaction of cationic drug amitriptyline hydrochloride with anionic and nonionic polymers. J. Mol. Liq..

[35] Paradiso P., Santos R., Horta R., Lopes J., Ferreira P., Colaço R. (2018). Formation of nanocrystalline tobermorite in calcium silicate binders with low C/S ratio. Acta Mater..

[36] Pellenq R.M., Lequeux N., Van Damme H. (2007). Engineering the bonding scheme in C–S–H: The iono-covalent framework. Cem. Concr. Res..

[37] Basquiroto de Souza F., Sagoe-Crentsil K., Duan W. (2022). A century of research on calcium silicate hydrate (C–S–H): Leaping from structural characterization to nanoengineering. J. Am. Ceram. Soc..

